# Parents’ Perceptions Regarding Needs and Readiness for Tele-Practice Implementation Within a Public Health System for the Identification and Rehabilitation of Children with Hearing and Speech–Language Disorders in South India

**DOI:** 10.3390/ijerph22060943

**Published:** 2025-06-16

**Authors:** Neethi Jesudass, Vidya Ramkumar, Shuba Kumar, Lakshmi Venkatesh

**Affiliations:** 1 Department of Audiology, Sri Ramachandra Faculty of Audiology and Speech Language Pathology, Sri Ramachandra Institute of Higher Education and Research (DU), Chennai 600116, Tamil Nadu, India; neethi.j@sriramachandra.edu.in; 2 India Alliance, Intermediate Fellow in Clinical and Public Health Research, Department of Audiology, Sri Ramachandra Faculty of Audiology and Speech Language Pathology, Sri Ramachandra Institute of Higher Education and Research (DU), Chennai 600116, Tamil Nadu, India; 3 SAMARTH, Chennai 600004, Tamil Nadu, India; shubakumar@samarthngo.org (S.K.); lakshmiv@sriramachandra.edu.in (L.V.); 4 Department of Speech Language Pathology, Sri Ramachandra Faculty of Audiology and Speech Language Pathology, Sri Ramachandra Institute of Higher Education and Research (DU), Chennai 600116, Tamil Nadu, India

**Keywords:** needs, readiness, tele-practice, public health system, community, focus group discussions, semi-structured interviews, community survey, parents’ perceptions

## Abstract

**Background:** Tele-practice, as an evidence-based practice, has gained momentum over the last two decades. However, routine clinical adoption is not spontaneous. Implementation science facilitates stakeholder engagement and the assessment of needs and plans. The study aims to assess the needs related to audiology and speech–language pathology services for children under six years of age and readiness for a tele-practice-based model of care for diagnostic and rehabilitation services among parents within the public sector in Tamil Nadu. **Methods:** A cross-sectional study design was used. The qualitative methods of focus group discussions and semi-structured interviews were conducted for parents of children with disabilities. A quantitative community survey was conducted on parents of children with no known disabilities. A deductive-inductive method of analysis was used. **Results:** Community survey responses were analyzed using percentage analysis. The results were classified based on the constructs of Bowen’s feasibility framework: demand/need for tele-practice, acceptability of tele-practice, and integration and practicality of tele-practice. Parents considered the existing services to be inadequate. Parents perceived tele-practice as beneficial, yet they felt a hybrid method would be more suitable, with sufficiently interspersed in-person visits. Parents believed that training and community awareness were necessary before implementing a technology-based model of services. **Conclusions:** The study’s findings guided the fine-tuning of the proposed comprehensive tele-practice model for hearing and speech–language services for children in this rural district.

## 1. Introduction

Children with disabilities “include those who have long-term physical, mental, intellectual or sensory impairments which in interaction with various barriers may hinder their full and effective participation in society on an equal basis” [1]. According to an analysis of secondary data from the National Family Health Survey-5 (NFHS-5) data, the prevalence of disability in India is 4.52% across all age groups. Hearing disability was reported to be 8% among children aged 0–14, while speech disability was 16.31% of the total population [2]. In Tamil Nadu, the proportion of individuals with disabilities in the total population is 1.64 per cent. Almost all childhood disabilities have a high risk of communication disorders. Early identification and intervention enhance the likelihood of a child experiencing fewer developmental delays and an overall improvement in quality of life [3].

Early childhood screening, assessment and rehabilitation schemes are implemented in developed countries like United States, United Kingdom, Australia [4,5]. However, there has been a challenge in implementing effective early identification and rehabilitation in the context of low- and middle-income countries (LMICs). Among the eight LMICs, at present, only Bangladesh and India from the South Asia region have national-level programs for the early identification of childhood disabilities [4].

India has 28 states, 640 districts and 640,932 villages [6,7]. India’s public health services are divided into three levels [8]. Where primary care is at the village level, secondary care is at district level and tertiary care is at state level. The primary level includes subcenters and primary health centers (PHCs). Community health centers (CHCs), taluk hospitals, and district general hospitals comprise the secondary level. The tertiary level includes state medical colleges and teaching hospitals.

In 2013, the Ministry of Health and Family Welfare (Indian government) launched the Rashtriya Bal Swasthya Karyakram (RBSK) at primary and secondary levels to screen children (0–18 years) for disabilities. The diagnostics and interventions in this program are provided through the district general hospital (GH)’s early diagnostic and intervention centers (EDC/DEIC) at the secondary level of the Indian public health care service structure [8]. The Ministry of Women and Child Development supports RBSK triage through pre-school teachers (Anganwadi). Through the Ministry of Social Justice and Empowerment, several national institutes for disabled people and District Differently Abled Welfare Offices were established to provide social welfare at the tertiary level of Indian public health care.

The Ministry of Education’s Samagra Shiksha Abhiyan (SSA) is a holistic, integrated school education program for pre-school to class 12 children; it provides high-quality education in an equitable and inclusive classroom. This scheme also includes preliminary childhood disability screening, and special educators provide special education to children with disabilities.

In 2007, the National Programme for Prevention and Control of Deafness (NPPCD) by GOI was instituted to enhance the implementation of hearing care services. There are other statewide schemes such as the centralized newborn hearing screening program in Kerala [9] or the cochlear implantation scheme in various states of India [10,11].

While the national programs and schemes have existed in India for over a decade, these initiatives are impacted by insufficient infrastructure, lack of proper referral systems, and unfilled positions of health care providers (HCPs) [12]. There is a stark shortage of HCPs that provide rehabilitation services, like audiologists, speech therapists, and special educators, in semi-urban and rural locations of the RBSK program [13]. India has 4.41 audiologist/SLPs per 100,000 people, according to the Rehabilitation Council of India annual report [14]. Most of the Indian population, especially in rural areas, receive health care services from unqualified providers [15]. Limited professional availability and the difficult commute to these facilities in urban and rural areas hinder service use, especially for families in remote or rural areas. Due to a shortage of trained health workers, rural health facilities struggle to staff specialists and HCPs [16].

Tele-practice may help address these issues [17]. Tele-practice has grown in health care and allied health services over the past decade. Tele-practice can optimize medical resource use, improve clinical diagnosis, treatment, and care, and expand health care access. Tele-practice has been used to fill gaps in childhood disability service delivery [18,19,20,21] and to support hearing and speech–language disorder rehabilitation. Tele-practice-based audiology services are mostly funded by research [22,23] and urban. The need is greatest for rural residents who rely heavily on public health services [24,25], especially LMICs. Telemedicine adoption and use in rural and remote areas has been difficult due to patient and clinician reluctance like telehealth activity was influenced by onboarding processes, clinician willingness to practice, strategic challenges and primary care activity [26]. Conducting a systematic need [27,28] and planning assessment with all key stakeholders [29] will promote reflective learning and a better understanding of the issues. Through systematic implementation science, its research can be translated into clinical use [30,31].

In Tamil Nadu, a southern state of India, a comprehensive tele-practice model for hearing and speech–language services for rural children in Tamil Nadu was developed to study the implementation feasibility of addressing service gaps for early identification and rehabilitation of children with hearing and speech language disorders in the public sector.

The aim of this study is to assess the needs and readiness for tele-practice implementation within a public health system for the identification and rehabilitation of children with hearing and speech–language disorders under six years of age. The study was created to understand the perceptions of parents of children with disabilities and parents of children with no known disabilities under six years of age regarding their need to access the public sector diagnostic and rehabilitation services and their readiness to accept a tele-practice model of care for children with disabilities in the state of Tamil Nadu (Southern India).

The findings from this tele-practice planning assessment were to guide the implementation of the proposed comprehensive tele-practice model for hearing and speech–language services.

## 2. Materials and Methods

The research study received approval from the Institutional Ethics Committee of Sri Ramachandra Institute of Higher Education and Research (Deemed to be University), in accordance with national ethical standards. The reference number is IEC-NI/19/NOV/71/90.

### 2.1. Selection of Study Site

Twelve districts were initially shortlisted among the thirty-eight districts in the state of Tamil Nadu based on the proportion of children under the age of six, distance from Chennai, area in square meters, socio-economic status and rural nature. Following the recommendations and permission received from the Office of the State Commissionerate for Welfare of the Differently Abled, Tamil Nadu, the study locations were chosen to be two rural districts, namely Ariyalur and Perambalur. These districts had nearly equivalent populations, areas, socio-economic conditions and were both rural in nature.

### 2.2. Study Design

**Figure 1:**
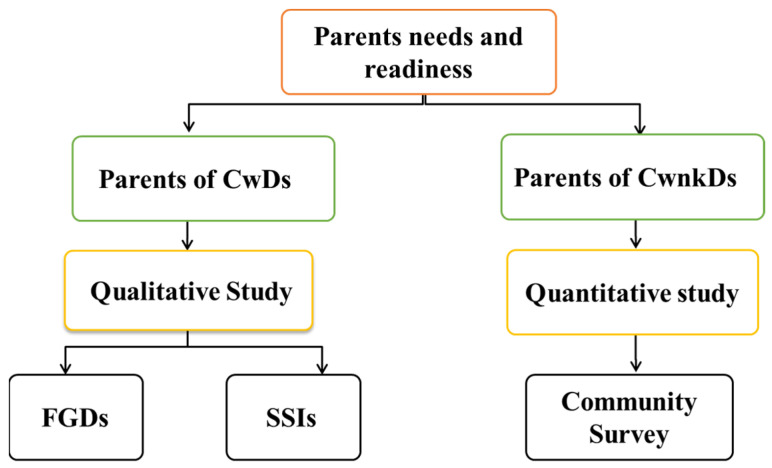
Schematic representation of the study design.

The study used a cross-sectional study design that included qualitative data from Focus Group Discussion (FGDs) and Semi-Structured Interviews (SSIs) for parents of Children with Disabilities (CwDs). A quantitative community survey involving a structured questionnaire was carried out with parents of Children with no known disabilities (CwnkDs) (Figure 1).

The Consolidated Criteria for Reporting Qualitative Research (COREQ) Checklist [32] was used to develop and report the qualitative study methods, context, findings, analysis and interpretations (Supplementary Materials S1).

### 2.3. Study Participants

#### Parents of Children Under Six Years of Age

Qualitative study with parents of CwDs (FGDs and SSIs)

The addresses of CwDs were first obtained from the government database for the two districts. All parents of CwDs in these districts were then invited to participate in the study with the help of field workers recruited for the study. The field workers visited the participant’s home and followed up through phone calls. Participants who provided written consent were invited to participate in an FGD or an SSI. Those who participated in the FGDs were not included in SSIs and vice versa. Purposive homogeneous sampling was used to select participants who share highly similar characteristics for the study [33]. Fathers and mothers of CwDs and fathers and mothers of CwnkDs, as well as their age and socioeconomic status were taken into account to maintain homogeneity.

Quantitative study with parents of CwnkDs (community survey)

The survey was conducted on parents of children less than six years of age with no known disabilities residing in the study area regarding their awareness, access to public sector services, need for diagnostic and rehabilitation services related to childhood disability and their readiness for the tele-practice model. Considering the fact that several childhood disabilities present with some form of speech and/or language disorder, parents of children with any disability were of interest. Since screening is an activity that involves the general public, parents of children with no known disability were also considered relevant. Parents were invited to participate in the study through the respective field workers in each district employed through the project. Based on the population sizes of the districts and the 95% confidence interval, the estimated sample size was 660 participants in each study site [34], adding up to a total required sample size of 1320 participants for the two districts.

A conceptual framework from previous research used to study the needs and readiness for tele-practice in rural areas for childhood communication disorders was adopted [35].

### 2.4. Data Collection Tools

#### 2.4.1. Qualitative Study—Guidelines

Based on Bowen’s framework [36], the guidelines for the FGDs and SSIs sought information about the following: the availability of current services for diagnosing and treating hearing and speech–language impairments; perceived need as well as the acceptability of tele-practice as an alternate option; parents’ readiness to accept and utilize technologies (such as cell phones, computers, and the internet); and feelings about possible challenges. The interview guides were refined and enhanced with information from each interview. Additionally, the guidelines for parents of CwDs were in English and Tamil, facilitating greater understanding (Supplementary Materials S2 and S3). The content, words, and relevance with the intended objectives of the guides were further reviewed by two experts who had expertise in qualitative research and tele-practice.

#### 2.4.2. For Quantitative Study—Survey Questionnaire

The survey questionnaire consisted of 20 items in English and Tamil that aimed to obtain information about parents’ awareness of childhood hearing and speech–language disorders and services, accessing services for speech–language and hearing disorders and acceptance for m-health/doorstep services (Supplementary Materials S4 and S5).

Face validation of the survey questionnaire was performed by two experts, one in social science and another in community-based services. Their suggestions were reviewed and incorporated into the final survey questionnaire, following which the questionnaire was pilot-tested among 22 mothers of children under six years of age residing in the Ariyalur and Perambalur districts. They provided feedback on the items’ meaningfulness, relevance and applicability, based on which the questionnaire was finalized.

The survey tool was subjected to test–retest reliability on the same 22 participants. The gap between the test and retest was 13 days. Statistical analysis was performed using ‘IBM SPSS statistics 22’ software to obtain intra-class correlation (ICC). Fifteen items had ICC greater than 0.9, indicating excellent reliability, and the remaining five items had ICC of 0.75 to 0.90, indicating good reliability. The survey tool was therefore considered reliable for data collection.

### 2.5. Data Collection

#### 2.5.1. Qualitative Study

**Figure 2:**
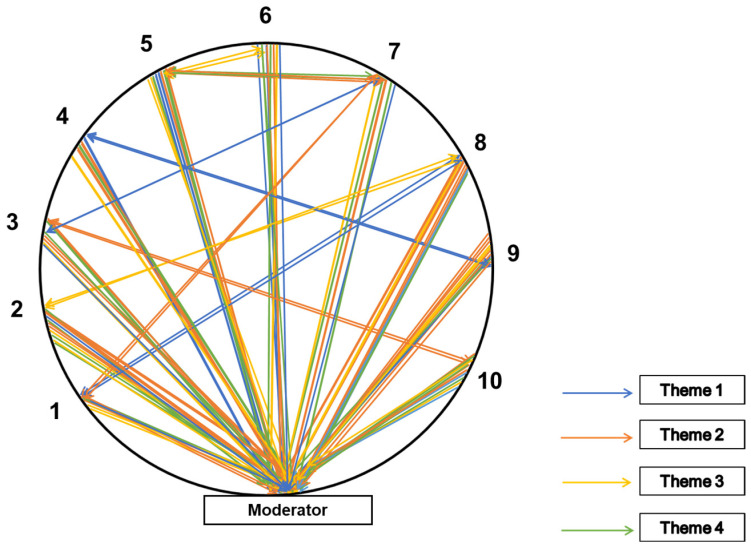
Schematic representation of the sociogram for FGD (10 Participants).

Prior to data collection, written informed consent was obtained from all participants. The investigator (a female audiologist and speech–language pathologist) underwent training in qualitative research before the study, and the research supervisor was already trained in qualitative research methods; one of the research advisors is a social scientist and master trainer for qualitative studies. In addition to audio recording of every FGD and SSI, a paper-based sociogram for FGD and field notes were also prepared. Sociograms are a valuable tool in qualitative research used to graphically represent group dynamics as well as to describe and interpret data from focus group discussions [37]. Sociograms provide a viable approach to enhance the methodological rigor of focus groups in health care research and complement content analysis [38]. The use of sociograms in the study encouraged all participants to participate in the discussion. Figure 2 is a schematic representation of a sociogram used during data collection in the focus group discussion. It was prepared as a paper-based tool to map participant responses. The figure illustrates how the moderator conducted the session, with the numbers indicating the number of participants in each group. The differently colored solid lines represent participants’ perceptions related to various emerging themes. The arrows pointing toward the participants represent questions from the moderator, whereas the arrows pointing toward the moderator indicate participants’ responses.

The FGDs and SSIs were conducted in special schools, non-governmental organization (NGO) offices, and trusts in the districts of Ariyalur and Perambalur, and a few SSIs were conducted in the participant’s homes in 2021. The FGDs were concluded within 45 min, whereas the SSIs were completed in approximately 30 min. Cross-case variance [39] and theoretical saturation [33,40] were utilized to evaluate the sufficiency of the gathered data.

#### 2.5.2. For Quantitative Study

A community survey was conducted among parents of CwnkDs. The investigator trained field workers from both districts to conduct community surveys. The investigator initiated the door-to-door survey in each district, and two field workers observed the survey process. These field workers are special educators working in special schools. The investigator provided field workers with instructions on how to ask each survey question. The investigator then observed the field workers surveying 20 participants. The community survey was conducted in Tamil through doorstep visits by a trained field worker. The field worker also contacted parents through village heads and school teachers in the community. Informed e-consent was sought from all participants before data collection.

### 2.6. Data Analysis

#### 2.6.1. For Qualitative Study

The audio recordings of Tamil transcripts of the FGDs and SSIs were translated to English and imported into NVivo software version 12. In the initial phase, a codebook was developed using a hybrid deductive–inductive analytical method guided by Bowen’s feasibility framework. Subsequently, using the thematic analytical approach described by Braun and Clark [41,42], data was familiarized through repeated readings of the transcripts. Data was then coded using the code book developed earlier to inductively add new codes based on new information gathered. Five transcripts were independently coded by the investigator and the research supervisor. The existing codebook was revised with the addition of codes derived inductively from the interviews. Any coding differences were resolved through discussion with the social scientist (research advisory member). The remaining transcripts were coded using the updated codebook. The significant patterns in the data that were pertinent to the research objectives were identified and categorized into themes using the coding categories.

As a result of this exercise, seven categories for parents of CwDs (Table 1) were identified. The categories went through an in-depth review and proceeded towards the theme development stage to assess the extent to which they described the data and answered the research questions. The described procedure was iterative in nature, with revisits to the transcripts and the assessment of the codes and sub-categories. Following this, two themes were identified among parents, which provided valuable insights into their needs and readiness to accept and adopt tele-practice-based services. Each theme was named and supported by relevant quotations extracted from the transcripts.

#### 2.6.2. Quantitative Study

All the community survey responses were translated to English and entered into a Microsoft Excel spreadsheet for the purpose of analysis in Microsoft for Windows. The survey responses were analyzed using percentage analysis.

## 3. Results

This section elaborates on the perceptions of parents of CwDs and parents of CwnkDs regarding their need for audiology and speech–language pathology services and their readiness to accept and adopt tele-practice-based services.

### 3.1. Participant Description

#### Parents of Children (with CwDs and CwnkDs)

In all, 61 parents of CwDs from both the districts participated in the FGDs and SSIs, of whom 33 were mothers in the age range of 25 and 37 years (average = 29.3) and 28 were fathers in the age range of 34 and 45 years (average = 39). The average (mean) age of parents (mothers and fathers) was 34 years. Few SSIs with fathers of CwDs were conducted in their residences because there were fewer than six participants to conduct an FGD.

In all, 1722 parents of CwnkDs from both districts participated in the community survey, of whom 1242 were mothers in the age range of 18 and 50 years (average = 29.02) and 480 were fathers in the age range of 20 and 45 years (average = 29.07). The average (mean) age of parents (mothers and fathers) was 29 years. Since trained field workers from the community conducted the survey, more data could be collected than the estimated sample size. The majority of mothers were homemakers, and few worked in farms as daily wage laborers, whereas the majority of fathers worked either in farms or in construction sites as daily wage laborers. The parent participant details are provided in Table 2.

### 3.2. Perceptions of Parents of CwDs and Parents of CwnkDs Regarding Their Need for Audiology and Speech–Language Pathology Services and Their Readiness to Accept and Adopt Tele-Practice-Based Services

The perceptions of parents of children with and with no known disabilities under the age of six are presented based on the constructs of Bowen’s framework, which guided this study. The need for tele-practice was assessed using the constructs of ‘demand/need’ for diagnostic and rehabilitation services and readiness to accept and adopt tele-practice was assessed using the constructs of ‘acceptability’, ‘integration’, and ‘practicality’. The constructs of integration and practicality were combined and presented as one theme, as there was a considerable overlap between these constructs.

#### 3.2.1. Need for Tele-Practice

Demand/need

(i) Parents’ perceptions of their need to access the public sector diagnostic and rehabilitation services with specific emphasis on audiology and speech–language pathology services

(i.a) Parents of CwDs

Parents were asked about their understanding and availability of facilities for speech, language and hearing problems. The possible barriers to accessing services and suggestions, if any, to improve the services were also probed. Parents of CwDs reported that no specialists were available in these districts, and they were generally unaware of the facilities available through the DDAW office and government facilities in these districts. A few of them went to the DDAW office to obtain a bus pass, a disability certificate, and hearing aids. Due to the unavailability of services, many traveled to other districts or visited private hospitals. Few individuals reported that testing facilities were available in government general hospitals, but the equipment was often under repair; consequently, they had received testing (screening) services for their child at government-sponsored camps.

Participants were generally not aware of the services provided through the Mobile Therapy Unit (MTU), which the government utilized in camps to conduct evaluations, provide speech and physiotherapy, and provide aids and appliances. Few of them reported that MTU was seen in camps, and special educators provided most speech therapy sessions.

Barriers to accessing services included poor transportation facilities to access the centers, lack of proper testing facilities, insufficient resources to repair machines, financial costs, waiting time for testing at the district general hospital, and need to travel long distances with the child to access services.

*“There are no testing facilities in the public sector in Ariyalur. We have to go to Thanjavur Trichy, Pondicherry”*

(Mothers of CwDs, FGDs).

*“Special educators provide speech therapy and not speech therapists”*

(Fathers of CwDs, FGDs).

*“If the facilities are provided nearby home, then it’s better”*

(Mothers of CwDs, FGDs).

*“As there is a greater number of children in need of service, the bigger organization should help them out. The help needed are specialists. Currently having financial issues as a daily wage laborer”*

(Fathers of CwDs, FGDs).

The parents’ suggestions for improving the quality of the service were to make testing facilities available near the district general hospital, provide speech therapy consistently, and increase the availability of specialists (audiologist/speech–language pathologist). 

(i.b) Parents of CwnkDs

**Figure 3:**
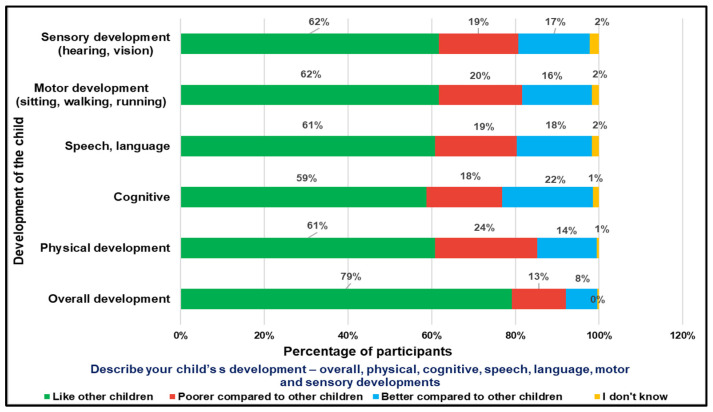
Parents’ of CwnkDs perceptions of their child’s development.

The survey’s first question on awareness about child development probed about the perceptions of parents of CwnkDs about their child’s development in various domains (Figure 3). On average, 60% of parents perceived that their child’s development was similar to that of other children in the community. However, around 19% of parents perceived that their child’s sensory and speech–language development was poorer compared to other children. And this, in a sample of 1722, amounts to approximately 300 children, which is a sizeable number. Furthermore, 1–2% of parents did not know how to assess their child’s growth and development.

A question on whether parents of CwnkDs had ever come across any child having speech, language or hearing difficulties was asked. It resulted in 61% of parents responding ‘No’, whereas 19% were unsure of what speech, language and hearing difficulties may seem like. And only 20% reported that they had seen a child with speech–language or hearing difficulty/disorder. These numbers indirectly also indicate lack of mainstreamed information and availability of services for childhood disability.

Availability of service centers and providers

**Figure 4:**
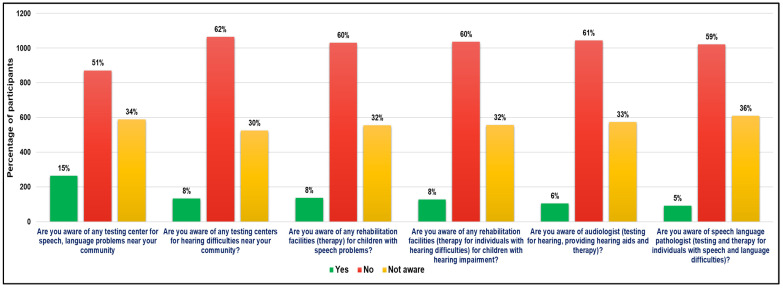
Parents’ of CwnkDs perceptions of the availability of service facilities and service providers.

The next six questions (questions 3 to 8) were asked to parents of CwnkDs to understand their perceptions of the availability of service facilities and providers. Survey responses probed parents’ awareness regarding diagnostic testing, rehabilitation centers and service providers for speech, language and hearing difficulties in their community (Figure 4). On average, 59% of parents reported that they did not come across any diagnostic and/ rehabilitation service centers/providers of speech–language and hearing disorders. Less than 8% of parents were aware of such service facilities and providers’ availability. Also, on an average 33%, which is a significant percentage, were not even aware of such services/ service providers. These findings indicate a considerable lack of availability of such services, implying the need for it.

**Figure 5:**
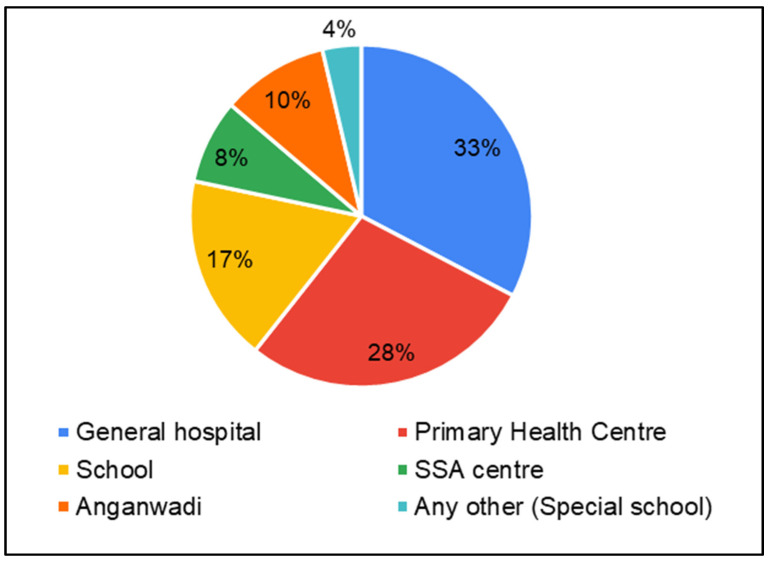
Parents of CwnkDs: availability of testing/rehabilitation facilities.

**Figure 6:**
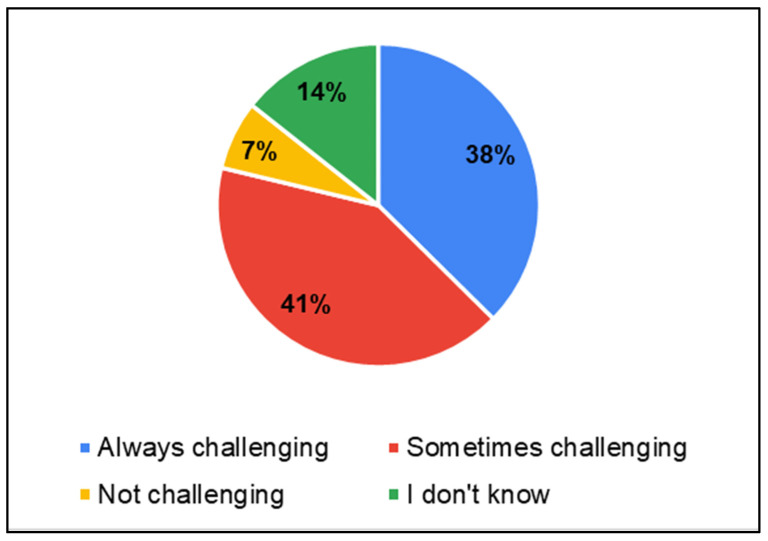
Parents of CwnkDs: availability of service providers.

Considering that in these districts, there was limited availability/accessibility of services specific to hearing, speech, and language disorders, parents were asked about public health service centers in general. This was asked to infer suitable service delivery points for hearing-, speech- and language-related services. Parents reported that services and HCPs for children were most available in general hospitals (GH—33%) followed by Primary Health Centers (PHC—28%) and schools (17%) (Figure 5). Parents predominantly perceived the availability of service providers as challenging (38% always challenging, 41% sometimes challenging) (Figure 6).

Accessibility of service centers and service providers

**Figure 7:**
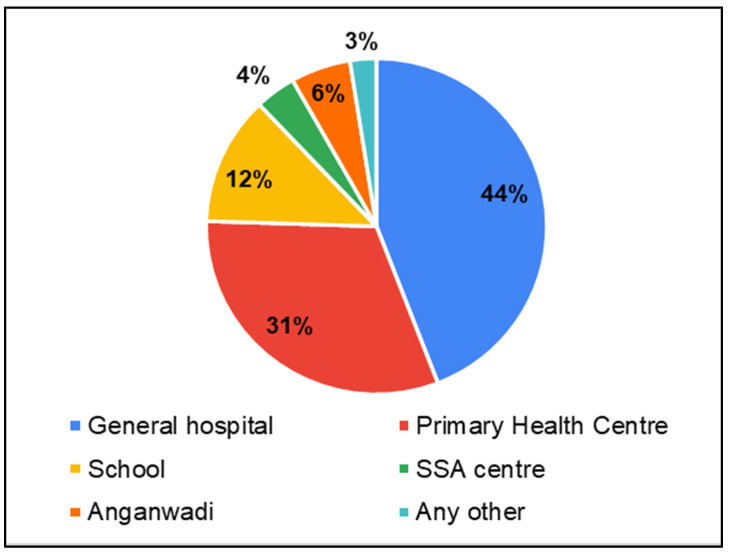
Parents of CwnkDs on accessibility: most difficult facilities to access health care services.

**Figure 8:**
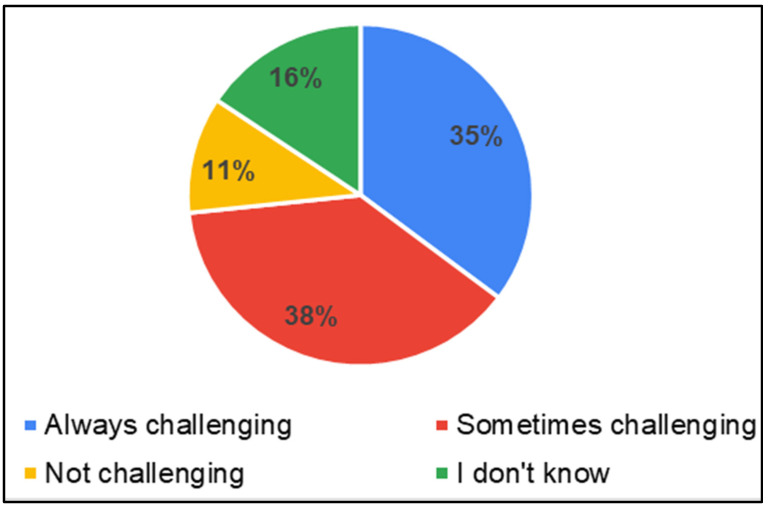
Parents of CwnkDs on accessibility: transport facilities to seek services.

**Figure 9:**
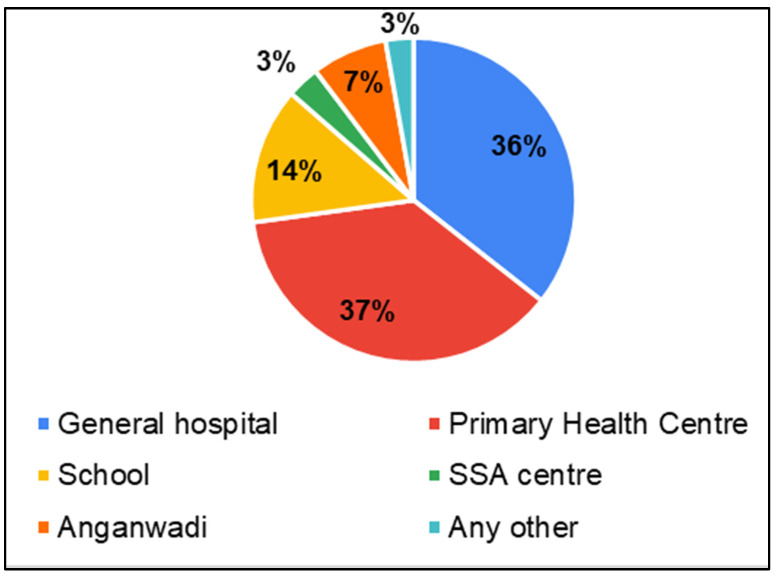
Parents of CwnkDs: limited time of service providers.

Though the services were available at GHs, it was challenging for parents to access the facilities at GHs. Access to GHs (44%) was the most difficult, followed by PHCs (31%), and schools (12%) (Figure 7). Further questions were asked regarding transport facilities/travel to seek health care for testing/therapy/any other service for children. Parents reported that accessing health care services was predominantly challenging (35%—always, 38% sometimes) due to inadequate transportation services (Figure 8). Though the service providers were available, their consultation time was limited in PHCs (37%) and GHs (36%) (Figure 9).

#### 3.2.2. Readiness to Accept and Adopt Tele-Practice Services

Acceptability of tele-practice service

(i) Parents acceptance of a tele-practice model of care for children with disabilities (CwDs)

(i.a) Parents of CwDs

Parents were asked about their familiarity with m-health services, their perceived comfort with using m-health to screen CwDs in the community, tele-diagnostics and tele-rehabilitation through MTU in a specific location closer to the children in need, and the availability of personnel to conduct tele-practice. Mothers who participated in the discussions either owned Android smart phones of their own or their spouses had one. They were aware of internet usage, yet only a few availed m-health services, even during the pandemic lockdowns, as they were not aware of a video consultation option with doctors.

*“At first, you can do it (speech therapy) in person and later you can do it through a laptop. Which makes the people trust you”*

(Fathers of CwDs, SSIs).

*“In Perambalur, most of them have smart phones for the purpose of studying (for children). Few of them have button phones (old model)*

(Mothers of CwDs, FGDs).

*“Nurses (Village health nurse) can be trained to do screening”*

(Mothers of CwDs, FGDs).

*“Anganwadi teachers can be trained for screening to reach all the children in the block”*

(Fathers of CwDs, SSIs).

They suggested that village health nurses or special educators could be trained to perform screenings in the community. They felt that the use of technology should not be spontaneous and suggested building awareness before deploying such (mobile-based screenings for hearing, speech, and language disabilities) services. They felt that in-person rehabilitation first, followed by tele-rehabilitation, was preferable, because they felt more comfortable with doctor consultations.

(i.b) Parents of CwnkDs

**Figure 10:**
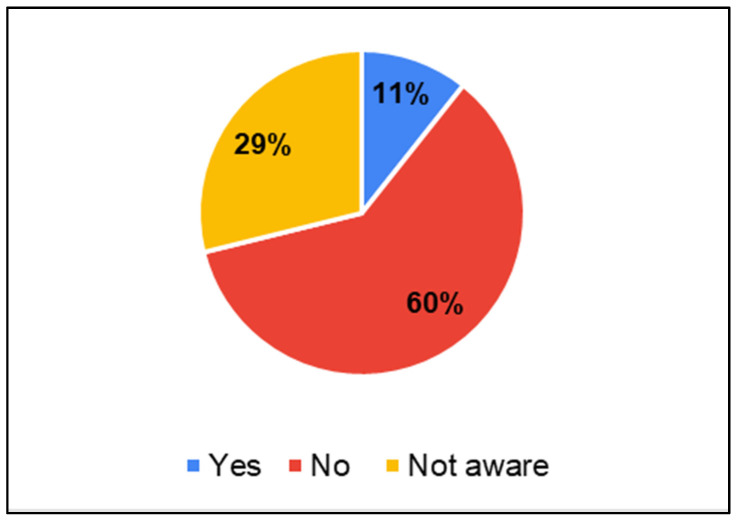
Parents of CwnkDs: mobile phone usage to seek health services.

**Figure 11:**
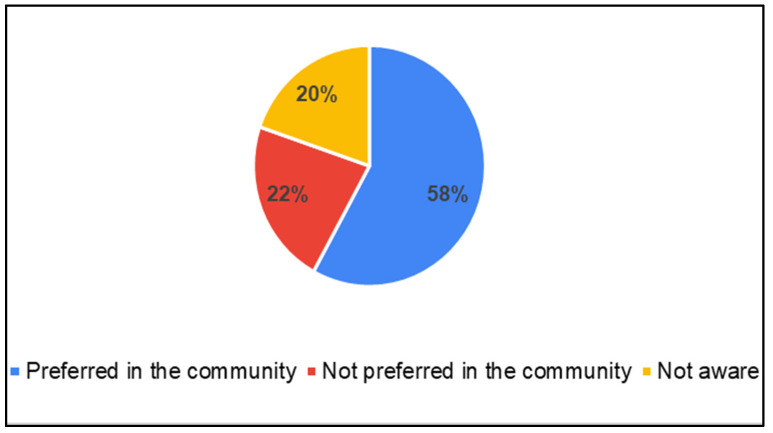
Parents of CwnkDs: doorstep services in the community.

**Figure 12:**
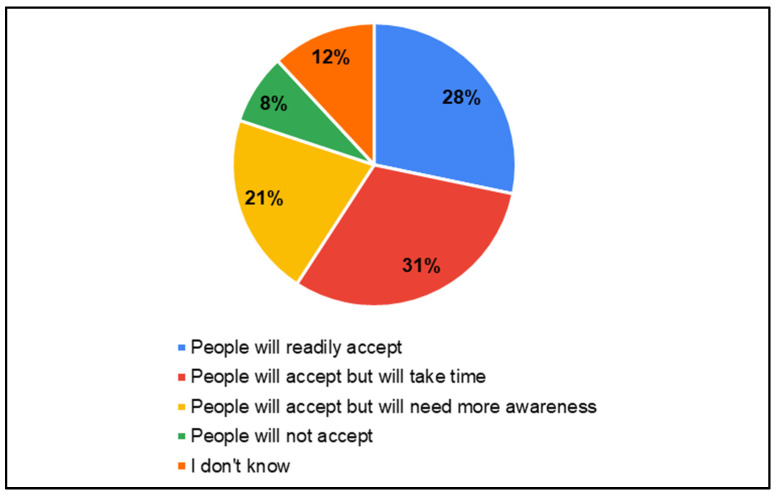
Parents of CwnkDs: mobile-phone based speech, language and hearing screening.

Parents of CwnkDs were asked about their usage of mobile phones to seek health services were asked. In order to verify the findings from Parents of CwDs, acceptance of door-step services, and integration of m-health tools was probed in the survey question. Similarly to Parents of CwDs, these parents also preferred door-to-door services for screening. But they had predominantly not sought services using mobile phones (60%) (Figure 10). A total of 20% of parents were not aware of mobile-based services (Figure 11). Parents believed mobile phone-based services could be provided to screen for childhood disabilities in the community (Figure 12).

Integration and practicality of tele-practice

(i) Parents’ acceptance of the tele-practice model of care for children with disabilities (CwDs)

(i.a) Parents of CwDs

Parents suggested using primary health centers or special schools at the block level to conduct tele-diagnosis and tele-rehabilitation. The fathers of CwDs felt that these services should be integrated with the hospitals in rural areas. They suggested making the MTU available at the block level so that more people could receive the services. The parents raised doubts about the availability of a suitable internet connection to support such services.

*“Village-wise service should be provided. With the help of a vehicle (mobile therapy unit). If the van is placed nearby then it will be beneficial as it’s difficult to carry the child to far places”*

(Fathers of CwDs, SSIs).

*“Services can be provided in school so everyone will come. GH, PHC and Panchayat are the common places for everyone to come”*

(Fathers of CwDs, SSIs).

*“Good awareness should be provided for the person with disability about the quality of service. Specialists should be available for differently abled people. If the service is provided then it will be helpful”*

(Fathers of CwDs, SSIs).

*“Services can be conducted in the school block-wise”*

(Mothers of CwDs, FGDs).

(i.b) Parents of CwnkDs

**Figure 13:**
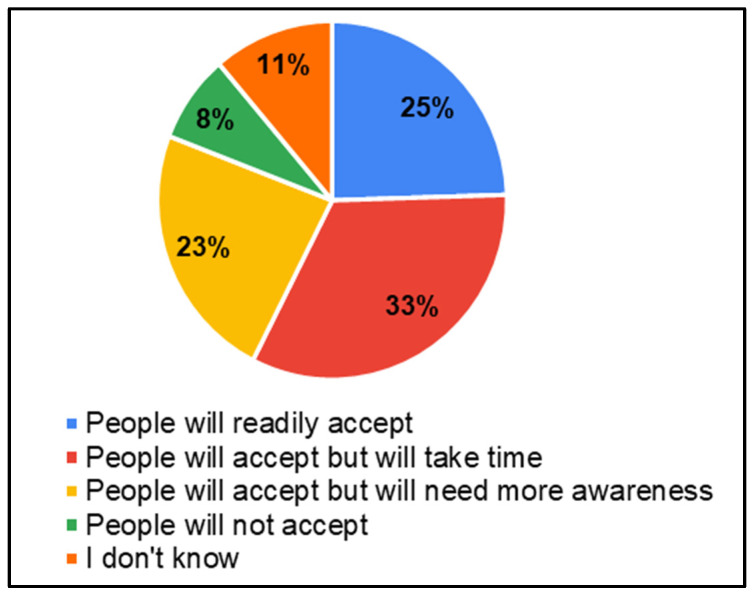
Parents’ of CwnkDs readiness—tele-diagnostics services.

**Figure 14:**
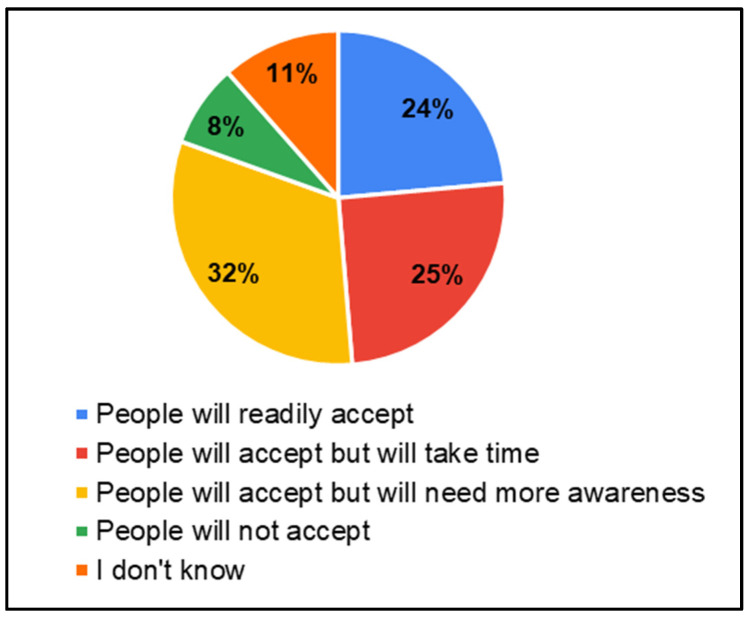
Parents’ of CwnkDs readiness—tele-rehabilitation services.

**Figure 15:**
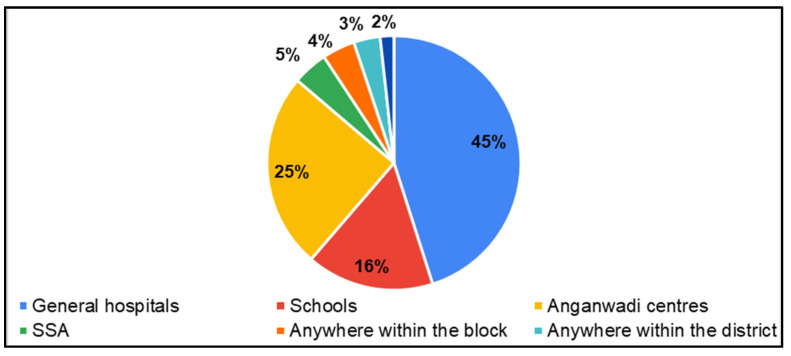
Parents of CwnkDs: suggested tele-practice delivery points for screening, diagnostic and rehabilitation services.

In the survey, parents of CwnkDs were asked about their acceptance of tele-practice in the community without expert testing centers and parents’ acceptance of hearing and speech–language testing procedures/rehabilitation (therapy) if provided through computers with internet near the community. Most parents accepted the tele-diagnostics (81%) and tele-rehabilitation (81%) services in the community, whereas few of the parents believed people would not accept tele-diagnostics (8%) (Figure 13) and tele-rehabilitation (8%) (Figure 14) services. The most frequently suggested locations for tele-diagnosis and tele-rehabilitation by parents of children with no known disabilities were the general hospitals, schools, and the Anganwadi centers (Figure 15).

## 4. Discussion

The study’s findings are discussed in detail under the sub-headings of (1) the need for diagnostic and rehabilitation services for hearing, speech and language disorders and (2) readiness to accept and adopt tele-practice-based diagnostic and rehabilitation services for hearing, speech and language disorders.

### 4.1. Need for Diagnostic and Rehabilitation Services for Hearing, Speech and Language Disorders

Parents of children under the age of six years of age emphasized the importance of having more service providers for hearing, speech, and language disorders, as well as improved access to services. Rural areas in India receive less health care services due to a shortage of staff and facilities [43]. Other low- and middle-income countries, such as Sub-Saharan Africa, had comparable issues with inadequate audiology and speech therapy services [44]. Bangladesh had only 9.4% physical therapists, 1.3% occupational therapists, 0.9% speech and language therapists, and 0.7% audiometrists per million people [45]. In developed countries like the US, the 2023 workforce estimate shows 57.7 per 100,000 population.

The quantitative and qualitative results from parents of CwnkDs and CwDs in this study showed the need for a comprehensive tele-practice model for speech, language, and hearing services in rural communities. In FGDs, SSIs, and surveys, parents highlighted a shortage of service providers and health care facilities as an important barrier. Parents in remote locations of Australia reported similar findings about a lack of access of audiological services [46].

In this study, CwD parents were unaware of the DDAW office, GH, and MTU health care services. The parents had to wait for the government’s free programs to undergo the testing. Parents of CwnkDs in this study were unaware of speech, language, and hearing difficulties. 1–2% of CwnkD parents did not know how to analyze or doubt their child’s difficulties. This is also apparent in a previous study conducted in India, which reported that parents were unaware of disabilities [47,48,49].

In this study, parents of CwDs reported significant challenges to receiving even the limited resources. Lack of public transportation, road connections, larger travel distances with the child, financial costs, and testing waiting times were the difficulties, whereas parents of CwnkDs reported inadequate transport facilities to access health care services. Australia had similar challenges with limited public transportation, poor road connectivity, and long rural travel times [50]. Parents or caregivers of CwDs in LMICs have reported barriers to the accessibility of medical or allied health sciences. Inability to recognize their children’s health needs, lack of doctors to provide caregivers with information, shortage of health care providers, poor access to specialists, and failure of health care providers to provide information or guidance on government financial aid were barriers to accessing health care in India [51]. South Africa and other LMICs have identified financial restrictions, problematic care networks and opportunity costs, community stigma and lack of safety, a lack of trust in services, inability to change, and self-stigmatization as care barriers [52]. The authors also noted everyday transportation issues and congested areas as barriers to care [53]. Insufficient health services, unsuitable design and facilities, inconvenient transportation, and financial difficulties were all reported in Thailand [54]. The above-mentioned studies show that adequate HCPs are available to provide health care services in high-income countries. The lack of HCPs, infrastructure, transportation, and financial resources to offer routine services limits health care access in LMICs like India, Bangladesh, and Sub-Saharan Africa. It is the same scenario for services for hearing and speech–language disabilities.

Parents of CwDs in the current study traveled to other districts or private hospitals due to the unavailability of public sector services. This was also evident in a previous study in India, where most people utilized private health care rather than public health care. This reliance on the private health sector is a contributing factor to the disparities in the health status of individuals and the access and utilization of health care facilities [55]. Parents of CwnkDs reported a lack of mainstreamed information and resources for childhood disabilities. These barriers typically occur in rural areas and are considered a barrier to accessing routine therapy services [56].

In the current study, parents suggested improving service quality, building testing facilities nearby, providing consistent speech–language therapy, increasing ASLP availability in these districts, raising awareness of hearing and speech–language difficulties, improving transport accessibility to health care services, and increasing service provider consultation time.

### 4.2. Readiness to Accept and Adopt Tele-Practice-Based Diagnostic and Rehabilitation Services for Hearing, Speech and Language Disorders

Parents of children reported readiness towards mobile/tablet-based screenings for hearing, speech, and language disorders. Parents emphasized the need for community-based services from health care providers in remote locations. Parents accepted tele-diagnosis but were concerned with tele-rehabilitation being the only service. They preferred a hybrid strategy for rehabilitation.

Tele-practice can help remote or low-resource locations build sustainable services when professional resources are limited [25,57]. Tele-practice has been successful in identifying and rehabilitating hearing and speech–language disabilities in various countries [58,59,60,61,62,63,64,65,66,67,68]. Smartphone and tablet-based preschool hearing screening [69,70] and video-otoscopy middle ear screening [71,72] have been investigated. M-health apps screen for communication disabilities during development [73].

Parents of CwDs and CwnkDs believed tele-practice would be beneficial and should be available to all community blocks. In a study conducted in rural New South Wales, Australia, the authors recommended place-based and person-centered strategies, outreach programs with individuals and local communities, support for families’ travel access to locally available services, and technology to improve service delivery [50]. In this study, CwDs and their families felt that block-level health care professionals’ community-based services were beneficial. Telehealth can improve patient–provider communication and access to health care, improving care quality [74]. In a recent study in Australia, hearing providers found that most participants were happy about post-pandemic tele-audiology adoption and preferred it in the future. Positive attitudes concerning future tele-audiology usage were observed [75]. Previous research in India and other low-middle income nations found gaps in awareness, information, and knowledge [48,49,76]. In the current study, parents suggested raising awareness before implementation, which was also supported by recent studies [77,78].

In the current study, stakeholders preferred a hybrid approach for tele-rehabilitation for speech, language and hearing services. Previous studies [21,67,79,80,81] suggest a hybrid approach for tele-rehabilitation based on successful outcomes. In US, the mobile therapy unit provided in-person and hybrid physiatry care to children with special needs [82]. A hybrid virtual care model was acceptable to parents, therapists, and physicians, as it maintained the quality of care and facilitated parent education.

The usage of tele-rehabilitation was found to be effective for allied-health sciences. During the COVID-19 pandemic in Italy, 80.5% of caregivers of children with neurodevelopmental impairments felt satisfied with tele-rehabilitation provided by physiotherapists, speech therapists, occupational therapists, developmental neuropsychologists, professional educators, and psychiatric rehabilitation therapists [80]. Tele-rehabilitation in physical therapy provides equivalent clinical outcomes to traditional face-to-face approaches [81]. Tele-rehabilitation was valued by Indian parents of children with cerebral visual impairment for consistent monitoring, access to professional services, reachable and convenient resources, expert guidance, tailored care and intervention, parental training, technological use, and parental empowerment [83].

Tele-practice for allied health services has been accepted and useful in several countries, according to the above studies. Task-shifting strategies for m-health services in LMICs have been effective for rural residents when professionals were unavailable.

Telehealth use, education, and consistent care models can improve distant patient care [80]. A recent international survey of audiologists found that during the COVID-19 pandemic, the perceived importance of telehealth increased significantly from 44.3 percent to 87.1 percent. Furthermore, the use of telehealth in the past (41.3%), current (61.9%), and expected (80.4%) has increased [79].

In the current study, parents of CwDs recommended block-level PHCs or special schools for tele-practice. They were concerned about internet capacity. Block-level MTUs were also suggested by parents of CwDs to increase service usage. Fathers of CwDs prioritized integrating services with hospitals to increase rural health care access. Parents of CwnkDs recommended GHs, Anganwadi schools, and schools for tele-practice delivery. 

Parents of this study reported inadequate services for hearing, speech, and language disorder in both districts. FGDs, SSIs, and community surveys show that access to services is limited and tele-practice is needed. Parents also expressed readiness for m-health services and tele-diagnostic services. However, they preferred a hybrid approach for tele-rehabilitation as in-person therapy facilitates rapport and eye-to-eye contact for communication, which are critical components for delivery of speech and language services. From the community survey, parents of CwnkDs also preferred block level services in both districts. Parents of CwDs suggested MTU can be used at the block level to provide services for hearing and speech–language disorders.

## 5. Conclusions

This study identified needs related to audiology and speech–language pathology services for children under six years of age, including training of qualified health care providers, an increase in health care providers at village or block levels, and enhanced diagnostic and rehabilitation services for children with disabilities. The mobile/tablet-based services at the door step, tele-diagnostic services, and preference for hybrid mode for tele-rehabilitation were the aspects of readiness for a tele-practice-based model of care for diagnostic and rehabilitation services identified by various stakeholders within the public sector in Tamil Nadu. This study identified several crucial practical and integration-related aspects, including the suitability of VHNs, SSA special educators and Anganwadi teachers as screening personnel, the suitability of mobile therapy vans for tele-practice, and the necessity for block-level services. The findings of this tele-practice planning assessment guided the fine-tuning of the proposed comprehensive tele-practice model for hearing and speech–language services for children in these rural districts. The knowledge gained will serve as the initial phase in planning the implementation of tele-practice in public sector health services.

### Study Outcomes

The findings of this study were submitted as a report to the office of State Commissionerate for Welfare of the Differently Abled, Tamil Nadu for future planning of services in the state, and particularly towards the planning of the TNRights Community-Based Rehabilitation Program, currently under pilot. The findings were also disseminated using folk art methods to the public in both the study districts.

## Figures and Tables

**Table 1:** CwDs: categories and themes.

Categories	Themes
Category 1: Availability	Parents of CwDs—demand or need
Category 2: Barriers and challenges in seeking health care	
Category 3: Satisfaction with quality of health care	
Category 4: Suggestions to improve quality of health care	
Category 5: Perceptions on use of technology or mobile phones	Parents of CwDs—acceptability and integration of tele-practice services
Category 6: Rehabilitation—acceptance of new services	
Category 7: Screening—acceptance of new services	

**Table 2:** Parent participant details in qualitative and quantitative studies.

FGDs:7 (*n* = 53 Participants) and SSIs: 8 (*n* = 8 Participants)		
Stakeholders	No. of FGDs/SSIs	No. of Participants (Distribution in Each Group)
Mothers of CwDs	4 FGDs	33 participants (14, 6, 7, 6 in each group)
Fathers of CwDs	3 FGDs	20 (8,6,6 in each group)
Fathers of CwDs	8 SSIs	8 participants (one to one interview)
Parent of CwnkDs	1722 1242—mothers and 480—fathers (1015 from Ariyalur district and 707 from Perambalur district)	

## Data Availability

The required data are available in the supplementary files. Any additional data are available upon request from the corresponding author.

## Supplementary Materials

The following supporting information can be downloaded at: https://www.mdpi.com/article/10.3390/ijerph22060943/s1, Materials S1: COREQ Checklist; Materials S2: FGD/SSI Guide and Probes for Parents of CwDs–English; Materials S3: FGD/SSI Guide and Probes for Parents of CwDs–Tamil; Materials S4: Community Survey Questionnaire in English; Materials S5: Community Survey Questionnaire in Tamil.

## Abbreviations

The following abbreviations are used in this manuscript:

FGD	Focus Group Discussion
SSI	Semi-Structured Interview
CwDs	Children with Disabilities
CwnkDs	Children with no known Disabilities
LMICs	Low- and Middle-Income Countries
EDC	Early Diagnostic Centre
GH	General Hospital
DDAW	District Differently Abled Welfare
EIC	Early Intervention Centre
ASLP	Audiologist and Speech–Language Pathologist
SSA	Samagra Shiksha Abhiyan
VHN	Village Health Nurse
DEIC	District Early Intervention Centre
