# Supplementary material for: Parents’ Perceptions Regarding Needs and Readiness for Tele-Practice Implementation Within a Public Health System for the Identification and Rehabilitation of Children with Hearing and Speech–Language Disorders in South India
Source: Int J Environ Res Public Health. 2025 Jun 16;22(6):943. doi: 10.3390/ijerph22060943 (PMC12193409; doi:10.3390/ijerph22060943)
Supplement: Supplementary file 1 [file ijerph-22-00943-s001.zip › ijerph-3610067-supplementary.pdf]

Supplementary Material S1: COREQ CHECKLIST

Consolidated criteria for reporting qualitative studies (COREQ): 32-item checklist

Please indicate in which section each item has been reported in your manuscript. If you do not feel an item applies to your manuscript, please enter N/A.

No.	Item	Description	Section
**Domain 1: Research team and reflexivity**			
Personal characteristics			
1.	Interviewer /Facilitator	Which author/s conducted the interview or focus group?	Data analysis
2.	Credentials	What were the researcher's credentials? *E.g. PhD, MD*	Front page
3.	Occupation	What was their occupation at the time of the study?	Methodology – data collection
4.	Gender	Was the researcher male or female?	Methodology – data collection
5.	Experience and training	What experience or training did the researcher have?	Methodology – data collection
Relationship with participants			
6.	Relationship established	Was a relationship established prior to study commencement?	NA
7.	Participant knowledge of the interviewer	What did the participants know about the researcher? *E.g. Personal goals, reasons for doing the research*	Methodology - study participants (consent form)
8.	Interviewer characteristics	What characteristics were reported about the interviewer/facilitator? *E.g. Bias, assumptions, reasons and interests in the research topic*	Methodology - study participants (consent form)

**Domain 2: Study design**			
Theoretical framework			
9.	Methodological orientation and theory	What methodological orientation was stated to underpin the study? *E.g. grounded theory, discourse analysis, ethnography, phenomenology, content analysis*	Methodology - conceptual framework
Participant selection			
10.	Sampling	How were participants selected? *E.g. purposive, convenience, consecutive, snowball*	Methodology – study participants
11.	Method of approach	How were participants approached? *E.g. face to-face, telephone, mail, email*	Methodology – study design
12.	Sample size	How many participants were in the study?	Methodology – study participants
13.	Non-participation	How many people refused to participate or dropped out? What were the reasons for this?	Two fathers of children with disabilities - refused to participate as the participants were not interested in the topic discussed.
Setting			
14.	Setting of data collection	Where was the data collected? *E.g. home, clinic, workplace*	Methodology - study participants
15.	Presence of non participants	Was anyone else present besides the participants and researchers?	N/A

16.	Description of sample	What are the important characteristics of the sample? *E.g. demographic data, date*	Results – participant's description
Data collection			
17.	Interview guide	Were questions, prompts, guides provided by the authors? Was it pilot tested?	Methodology - data collection tools
18.	Repeat interviews	Were repeat interviews carried out? If yes, how many?	No
19.	Audio/visual recording	Did the research use audio or visual recording to collect the data?	Methodology - data collection
20.	Field notes	Were field notes made during and/or after the interview or focus group?	Analysis - transcripts
21.	Duration	What was the duration of the interviews or focus group?	Methodology - data collection
22.	Data saturation	Was data saturation discussed?	Methodology - data collection
23.	Transcripts returned	Were transcripts returned to participants for comment and/or correction?	None
**Domain 3: analysis and findings**			
Data analysis			
24.	Number of data coders	How many data coders coded the data?	Data analysis
25.	Description of the coding tree	Did authors provide a description of the coding tree?	Data analysis
26.	Derivation of themes	Were themes identified in advance or derived from the data?	Data analysis section

27.	Software	What software, if applicable, was used to manage the data?	Data analysis section
28.	Participant checking	Did participants provide feedback on the findings?	Yes
Reporting			
29.	Quotations presented	Were participant quotations presented to illustrate the themes / findings? Was each quotation identified? *E.g. Participant number*	*Results - Quotes*
30.	Data and findings consistent	Was there consistency between the data presented and the findings?	Results
31.	Clarity of major themes	Were major themes clearly presented in the findings?	Results
32.	Clarity of minor themes	Is there a description of diverse cases or discussion of minor themes?	Results

Supplementary Material S2: FGD/SSI GUIDE AND PROBES FOR PARENTS OF CwDs - ENGLISH

Demand/Need

1. Who and what facilities are available to identify speech, language and hearing problems in community?
Probes:
   a) Diagnostics services for 0-6 years CWD in the community?
   b) Who are involved? Human resources-professional versus others
   c) Time travel by you
   d) Time taken from suspect to confirmation
   e) Testing process

2. Who and What facility are available for rehabilitation with respect to speech language and hearing problems?
Probes:
MTU - From our interactions with DDAWOs and professionals in state commissioner office etc, we learnt that the govt has started MTU to help children get therapy and aids and appliances at their homes.

3. What about accessibility for testing?

4. What about accessibility for rehabilitation?
Probes:
   a) Human resources,
   b) Time management,
   c) Travel access
   d) Patient benefit – improvement in hearing and speech language abilities shown after therapy,
   e) Therapy consistency
   f) patient- economic
   g) MTU - from our interactions with ddawos and professionals in state commissioner office etc, we learnt that the govt has started mtu to help children get therapy and aids and appliances at their homes.

Probes -What are the barriers/challenges in seeking the health care?
Probes:
   a) Travel access
   b) Transport (bus/vehicle)
   c) Economic
   d) Time (to get services)
   e) Doctor’s availability
   f) Satisfaction with quality of healthcare

5. Do you have any suggestion on how to improve currently available services?

Probes:

a) Improving access/proximity
b) Improving consistency of service
c) Quality of service
d) Availability of service
e) Best human resources (SSA, ASLP, Anganwadi/balwadi workers)
f) Facilities
a) If we have to help children get speech therapist services at their homes or nearest to their homes, how can it be done?

6. Can you describe the ways in which mobiles/ tablets/ internet/ computers are used currently for receiving any services for disabilities in the community?

Probes:

a. Comfort of use for provider
b. Comfort of patients in using
c. Socio-cultural influences in accepting ICT

Readiness

7. We are developing screening devices to test children. In your opinion who will be the best person to conduct the test at last mile service (best reach)

Probes:

a) Their capacity for using technology,
b) Skills,
c) Readiness,
d) Current use of mobiles/internet/computers for health care

8. After screening, we will know who needs more services, and these children can be tested by audiologist from GH/ DDAWO office by sending testing equipment in the MTU to a particular location nearer to the location of children who need testing. And testing can be done using internet.

What is your opinion on this plan? Do you see any problems? Is there a better way to do this?

Probes: MTU, any physical fixed spaces in villages for last mile, what level - village, panchayat?

9. Once identified, we thought they can get certificate at DDAWO, and then for therapy, we can again provide through video call (in the presence of SSA worker/Anganwadi/balwadi workers) and give home plan. Monthly once the speech therapist can see child and give goals to mother.
Is there a better way to do this? Or what do you feel about this?
Probes:

a. Impact on time management
b. HR utilised
c. Usefulness and effectiveness of such services in bringing outcomes in children
d. Frequency of rehabilitation,
e. Monitoring of progress

Supplementary Material S3: FGD/SSI GUIDE AND PROBES FOR PARENTS OF CwDs - TAMIL

Demand/Need

1. இந்த சமூகத்துல காது பேச்சு மற்றும் மொழி குறைபாடுகள் உள்ள குழந்தைகளுக்கு யாரு பரிசோதனைகள் செய்கிறார்கள்?
   இந்த சமூகத்துல காது பேச்சு மற்றும் மொழி குறைபாடுகள் உள்ள குழந்தைகளை கண்டறிய என்ன வசதிகள் இருக்கிறது?
   ஆய்வுகள்:
   a. சமூகத்தில் குறைபாடுகள் உள்ள 0-6 வயது குழந்தைகளுக்கான குறைபாடுகள் கண்டறிதல் சேவைகள்
   b. சம்பந்தப்பட்டவர்கள் யார்? சுகாதார நிபுணர்கள் மற்றும் பிறர்
   c. உங்களுக்கான பயண நேரம்
   d. பரிசோதனையில் இருந்து குறைபாடுகளை உறுதிப்படுத்தும் நேரம்
   e. பரிசோதனை செயல்முறை
2. இந்த சமூகத்துல காது பேச்சு மற்றும் மொழி குறைபாடுகள் உள்ள குழந்தைகளுக்கு யாரு சேவை/பேச்சு பயிற்சி வழங்குகிறார்கள்?
   இந்த சமூகத்துல காது பேச்சு மற்றும் மொழி குறைபாடுகள் உள்ள குழந்தைகளுக்கு பயிற்சி பெற என்ன வசதிகள் இருக்கிறது?
   ஆய்வுகள்:
   நாங்க DDAWOs அலுவகத்தில் இருக்கிறவர்களிடம் (DDAWOs and professionals in state commissioner office) பேசினோம். அப்போ அரசாங்கம் (Government), குழந்தைகளுக்கு அவர்களின் வீடுகளில் சிகிச்சை மற்றும் உபகரணங்களைப் பெற உதவும் வகையில், மொபைல் தெரபி யூனிட்டை (MTU) அரசாங்கம் தொடங்கியுள்ளது என்பதை அறிந்தோம்.
3. இங்க குறைபாடுகள் உள்ள குழந்தைகளுக்கு பரிசோதனை கிடைக்கிறது பற்றி உங்கள் கருத்துக்கள் என்ன?
4. இங்க குறைபாடுகள் உள்ள குழந்தைகளுக்கு சேவை/பேச்சு பயிற்சி கிடைக்கிறது பற்றி உங்கள் கருத்துக்கள் என்ன?
   ஆய்வுகள்:
   a. மனித வளம்
   b. நேரத்தை நிர்வகித்தல்
   c. பயண அணுகல்

d. குழந்தைகளின் நன்மை - சிகிச்சைக்குப் பிறகு காட்டப்படும் கேட்கும் மற்றும் பேச்சு மொழி திறன்களில் முன்னேற்றம்
e. சிகிச்சை நிலைத்தன்மை
f. குழந்தைகளின் பெற்றோர் – பொருளாதாரம்
g. நாங்க DDAWOs அலுவகத்தில் இருக்கிறவர்களிடம் (DDAWOs and professionals in state commissioner office) பேசினோம். அப்போ அரசாங்கம் (Government), குழந்தைகளுக்கு அவர்களின் வீடுகளில் சிகிச்சை மற்றும் உபகரணங்களைப் பெற உதவும் வகையில், மொபைல் தெரபி யூனிட்டை (MTU) அரசாங்கம் தொடங்கியுள்ளது என்பதை அறிந்தோம்.

ஆய்வுகள்:
காது பேச்சு மற்றும் மொழி குறைபாடுகளுக்கு பரிசோதனை மற்றும் பயிற்சி பெறுவதற்கு சிரமம் இருக்கிறதா?
ஆய்வுகள்:

a. பயண அணுகல்
b. போக்குவரத்து (பஸ்/வாகனம்)
c. பொருளாதாரம்
d. நேரம் (சேவைகளைப் பெற)
e. மருத்துவர்கள் இருப்பது
f. சுகாதாரத் தரத்தில் திருப்தி

5. இப்போ இருக்கிற மருத்துவ வசதியை மேம்படுத்த உங்க ஆலோசனை சொல்லுங்கள்?
ஆய்வுகள்:
    a. சுகாதாரத்திற்கான அணுகல்/அருகில் மேம்படுத்துதல்
    b. சேவையின் நிலைத்தன்மையை மேம்படுத்துதல்
    c. சேவை தரம்
    d. சேவை கிடைப்பது
    e. சிறந்த மனித வளங்கள் (SSA, ASLP, அங்கன்வாடி/பால்வாடி பணியாளர்கள்)
    f. வசதிகள்
    g. குழந்தைகளின் வீடுகளிலோ அல்லது அவர்களின் வீட்டிற்கு அருகாமையிலோ பேச்சு சிகிச்சையாளர் சேவைகளைப் பெற நாம் உதவ வேண்டும் என்றால், அதை எப்படிச் செய்ய முடியும்?

6. கைபேசி அல்லது மடிக்கணனி மூலமாக எவ்வாறு குறைபாடு உள்ள குழந்தைகளுக்கு சேவைகள் தற்போது அழிக்கப்படுகிறது? அதை விவரமாக சொல்லுங்கள்.
   ஆய்வுகள்:
   a. வழங்குனருக்கான பயன்பாட்டின் வசதி
   b. உபயோகிப்பதில் குழந்தைகளின்/பெற்றோர் வசதி
   c. தகவல் தொடர்பு தொழில்நுட்பத்தை ஏற்றுக்கொள்வதில் சமூக-கலாச்சார தாக்கங்கள்

Readiness

7. நாங்க திரையிடல் சோதனை (screening test) பண்றதுக்கு ஒரு சாதனம் (device) செய்கிறோம். இந்த சாதனம் (device) யாரு பயன் படுத்தினால் எல்லாருக்கும் சோதனை பண்ண முடியும்?
   ஆய்வுகள்:
   a. தொழில்நுட்பத்தைப் பயன்படுத்துவதற்கான திறன்
   b. திறமைகள்
   c. தயார்நிலை
   d. சுகாதாரப் பாதுகாப்புக்காக
   e. கைபேசி/இணையம்/கணினிகளின் தற்போதைய பயன்பாடு

8. திரையிடல் சோதனைக்குப் பிறகு, யாருக்கு கூடுதல் சேவைகள் தேவை என்பதை நாங்கள் அறிவோம், மேலும் இந்த குழந்தைகளை GH/ DDAWO அலுவலகத்திலிருந்து ஆடியோலாஜிஸ்ட் (Audiologist) மூலம் MTU இல் உள்ள சோதனை உபகரணங்களை சோதனை தேவைப்படும் குழந்தைகளின் இருப்பிடத்திற்கு அருகில் உள்ள ஒரு குறிப்பிட்ட இடத்திற்கு அனுப்புவதன் மூலம் சோதிக்க முடியும். மேலும் இணையத்தைப் பயன்படுத்தி சோதனை செய்யலாம்.
   இந்த திட்டம் பற்றி உங்கள் கருத்து என்ன? நீங்கள் ஏதேனும் பிரச்சனைகளைப் பார்க்கிறீர்களா? இதைச் செய்ய சிறந்த வழி இருக்கிறதா?
   ஆய்வுகள்:
   மொபைல் தெரபி யூனிட், கடைசி மைலில் உள்ள கிராமங்களில் ஏதேனும் நிலையான இடங்கள், எந்த நிலை - கிராமம், பஞ்சாயத்து?

9. அடையாளம் காணப்பட்டதும், அவர்கள் DDAWO இல் சான்றிதழைப் பெறலாம் என்று நினைத்தோம், பின்னர் சிகிச்சைக்காக, மீண்டும் வீடியோ அழைப்பு மூலம் (SSA பணியாளர்/ அங்கன்வாடி/ பால்வாடி பணியாளர்கள் முன்னிலையில்) வீட்டுத் திட்டத்தை வழங்கலாம். மாதத்திற்கு ஒருமுறை பேச்சு சிகிச்சையாளர் குழந்தையைப் பார்த்து தாய்க்கு இலக்குகளை வழங்க முடியும்.
   இதைச் செய்ய சிறந்த வழி இருக்கிறதா? அல்லது இதைப் பற்றி நீங்கள் என்ன நினைக்கிறீர்கள்?
   ஆய்வுகள்:
   a. நேர மேலாண்மை காரணமாக பாதிப்பு
   b. மனித வளம் பயன்படுத்தப்பட்டது
   c. குழந்தைகளில் விளைவுகளைக் கொண்டுவருவதில்
   d. சேவைகளின் பயன் மற்றும் செயல்திறன்
   e. எத்தனை முறை சிகிச்சை
   f. முன்னேற்றத்தை கண்காணித்தல்

Supplementary Material S4: COMMUNITY SURVEY QUESTIONNAIRE IN ENGLISH

Demand/Need

Awareness about child development and services:

1. Describe your child's s development

1.1 Overall

1. Like other children
2. Poorer compared to other children
3. Better compared to other children
4. I don't know

1.2 Physical development

1. Like other children
2. Poorer compared to other children
3. Better compared to other children
4. I don't know

1.3 Cognitive

1. Like other children
2. Poorer compared to other children
3. Better compared to other children
4. I don't know

1.4 Speech, language

1. Like other children
2. Poorer compared to other children
3. Better compared to other children
4. I don't know

1.5 Motor development (Example: sitting, walking, running)

1. Like other children
2. Poorer compared to other children
3. Better compared to other children
4. I don't know

1.6 Sensory development (Example: hearing, vision)

1. Like other children
2. Poorer compared to other children
3. Better compared to other children
4. I don't know

2. Have you come across any child having speech, language and hearing difficulties?

a. Yes b. No c. Not sure

3. Are you aware of any testing centre for speech, language problems near your community

a. Yes b. No c. Not aware

If yes, give details…………

4. Are you aware of any testing centres for hearing difficulties near your community?

a. Yes b. No c. Not aware

If yes, give details…………

5. Are you aware of any rehabilitation facilities (speech therapy) for children with speech problems?

a. Yes b. No c. Not aware

If yes, give details…………

6. Are you aware of any rehabilitation facilities (therapy for individuals with hearing difficulties) for children with hearing impairment?

a. Yes b. No c. Not aware

If yes, give details…………

7. Are you aware of audiologist (testing for hearing, providing hearing aids and therapy)?

a. Yes b . No c. Not aware

If yes, give details…………

8. Are you aware of speech language pathologist (testing and therapy for individuals with speech and language difficulties)?

a. Yes b. No c. Not aware

If yes, give details…………

<u>Demand/Need</u>

Accessing services for speech-language and hearing disorders:

9. Accessibility with respect to transport facilities/travel to seeking health care (testing/therapy/any other service) for children is ….

a. Always challenging
b. Sometimes challenging
c. Not challenging
d. I don't know

10. Availability of child related services (for testing/therapy/any other service)

a. General hospital
b. Primary Health Centre
c. School
d. SSA centre
e. Anganwadi
f. Any other

11. Accessibility is most difficult to the following child related service providers (for testing/therapy/any other service)

a. General hospital
b. Primary Health Centre
c. School
d. SSA centre
e. Anganwadi
f. Any other

12. Availability of service providers (for testing/therapy/any other service) is ….

a. Always challenging
b. Sometimes challenging
c. Not challenging
d. I don't know

13. Availability of service providers (consultation for testing/therapy/any other service) is best at

a. General hospital
b. Primary Health Centre
c. School
d. SSA centre
e. Anganwadi
f. Any other

14. There is limited availability (consultation time) of service providers at

a. General hospital
b. Primary Health Centre
c. School
d. SSA centre
e. Anganwadi
f. Any other

Readiness

Acceptability and Integration

Acceptance for m-health/ doorstep services:

15. Doorstep childhood healthcare services (for testing/therapy/any other service) are

a. Preferred in the community
b. Not preferred in the community. If not preferred, provide details…………..
c. Not aware

16. Are mobile phones used in your community for receiving any health care services (for testing/therapy/any other service)?

a. Yes, give details   b. No   c. Not aware

17. If mobile phone-based services (for testing/therapy/any other service) are provided to screen childhood disability in the community

a. People will readily accept
b. People will accept but will take time
c. People will accept but will need more awareness
d. People will not accept
e. I don't know

Acceptance for tele-practice services:

18. In the absence of any expert testing centres, if hearing and speech, language testing procedures are provided through computers with internet near the community?

a. People will readily accept
b. People will accept but will take time
c. People will accept but will need more awareness
d. People will not accept
e. I don't know

19. In the absence of any expert rehabilitation centres, if rehabilitation (therapy) for hearing and speech-language is provided through computers with internet near the community.

a. People will readily accept
b. People will accept but will take time
c. People will accept but will need more awareness
d. People will not accept
e. I don't know

20. If diagnostic and rehabilitation (therapy) are provided at community, which is the most suitable place for such services

a. General hospitals
b. Schools
c. Anganwadi centres
d. SSA
e. Anywhere within the block
f. Anywhere within the district
g. Major cities nearby

Supplementary Material S5: COMMUNITY SURVEY QUESTIONNAIRE IN TAMIL

கணக்கெடுப்பு (survey) பற்றியவிளக்கம்:

இந்த கணக்கெடுப்பு, கிராமப்புற சமூகங்களில் செவிப்புலன், பேச்சு-மொழிகுறைபாடுகளுக்கான நோயறிதல் மற்றும் மறுவாழ்வு சேவைகள்தொடர்பான குறைபாடுகள் இல்லாத குழந்தைகளின் பெற்றோரின் கருத்துக்களைப் பற்றியது.

நீங்கள் இந்த கருத்துக்கணிப்பில் பங்கேற்க விரும்புகிறீர்களா?

a. ஆம்

b. இல்லை

<u>கணக்கெடுப்பு (Survey)</u>

பங்கேற்பாளரின் பெயர்:

பங்கேற்பாளரின் வயது:

உங்களுக்கு எத்தனை குழந்தைகள்? _____
முதல் குழந்தையின் வயது என்ன? ______
இரண்டாவது குழந்தையின் வயது என்ன? _______
மூன்றாவது குழந்தையின் வயது என்ன? ________
மூன்று குழந்தைகளுக்கு மேல் இருந்தால், குறிப்பிடவும்
____

நீங்கள் எந்த மாவட்டத்தைச் சேர்ந்தவர்?
a. அரியலூர்
b. பெரம்பலூர்

அரியலூர் மாவட்டத்தில் எந்த தொகுதி?
a. அரியலூர்
b. திருமானூர்
c. செந்துறை
d. ஜெயங்கொண்டம்
e. ஆண்டிமடம்
f. டி.பாலூர்

பெரம்பலூர் மாவட்டத்தில் எந்த தொகுதி?
a. பெரம்பலூர்
b. வேப்பந்தட்டை
c. வேப்பூர்
d. ஆலத்தூர்

1. உங்கள் குழந்தையின் வளர்ச்சியை விவரிக்கவும்
1.1 ஒட்டுமொத்த வளர்ச்சி
a. மற்ற குழந்தைகளைப் போல்
b. மற்ற குழந்தைகளுடன் ஒப்பிடும்போது குறைவான வளர்ச்சி
c. மற்ற குழந்தைகளுடன் ஒப்பிடும்போது சிறந்த வளர்ச்சி
d. எனக்கு தெரியாது
1.2 உடல் நல வளர்ச்சி
a. மற்ற குழந்தைகளைப் போல்
b. மற்ற குழந்தைகளுடன் ஒப்பிடும்போது குறைவான வளர்ச்சி
c. மற்றகுழந்தைகளுடன் ஒப்பிடும்போது சிறந்த வளர்ச்சி
d. எனக்கு தெரியாது
1.3 அறிவாற்றல்
a. மற்ற குழந்தைகளைப் போல்
b. மற்ற குழந்தைகளுடன் ஒப்பிடும்போது குறைவான வளர்ச்சி
c. மற்ற குழந்தைகளுடன் ஒப்பிடும்போது சிறந்த வளர்ச்சி
d. எனக்கு தெரியாது
1.4 பேச்சு, மொழி
a. மற்ற குழந்தைகளைப் போல்
b. மற்ற குழந்தைகளுடன் ஒப்பிடும்போது குறைவான வளர்ச்சி
c. மற்ற குழந்தைகளுடன் ஒப்பிடும் போது சிறந்த வளர்ச்சி
d. எனக்கு தெரியாது
1.5 செயல்பாட்டு திறன்
(உதாரணம்: உட்கார்ந்து, நடப்பது, ஓடுவது)
a. மற்ற குழந்தைகளைப் போல்
b. மற்ற குழந்தைகளுடன் ஒப்பிடும்போது குறைவான வளர்ச்சி
c. மற்ற குழந்தைகளுடன் ஒப்பிடும்போது சிறந்த வளர்ச்சி
d. எனக்கு தெரியாது

1.6 புலன்கள் சார்ந்த வளர்ச்சி (உதாரணம்: கேட்பது, பார்ப்பது)
a. மற்ற குழந்தைகளைப் போல்
b. மற்ற குழந்தைகளுடன் ஒப்பிடும்போது குறைவான வளர்ச்சி
c. மற்ற குழந்தைகளுடன் ஒப்பிடும்போது சிறந்த வளர்ச்சி
d. எனக்கு தெரியாது

2. பேச்சு, மொழி சிரமம் உள்ள குழந்தைகளை நீங்கள் கேள்விப்பட்டிருக்கிறீர்களா?
a. ஆம் b. இல்லை c. உறுதியாகதெரியவில்லை

3. உங்கள் சமூகத்திற்கு அருகிலுள்ளபேச்சு, மொழி பிரச்சனைகளுக்கான ஏதேனும் பரிசோதனைமையங்கள் உங்களுக்குத் தெரியுமா?
a. ஆம் b. இல்லை c. அறிந்திருக்கவில்லை
ஆம் எனில், விவரங்களைத் தரவும்
____

4. உங்கள் சமூகத்திற்கு அருகில் காதுகேளாமைக்கான ஏதேனும் பரிசோதனை மையங்கள் உங்களுக்குத் தெரியுமா?
a. ஆம் b. இல்லை c. அறிந்திருக்கவில்லை
ஆம் எனில், விவரங்களைத்தரவும்
____

5. பேச்சு, மொழி குறைபாடுள்ள குழந்தைகளுக்கான மறுவாழ்வு வசதிகள் (பேச்சு பயிற்சி) பற்றி உங்களுக்கு தெரியுமா?
a. ஆம் b. இல்லை c. அறிந்திருக்கவில்லை
ஆம் எனில், விவரங்களைத்தரவும்
____

6. செவித்திறன் குறைபாடுள்ள குழந்தைகளுக்கான மறுவாழ்வு வசதிகள் (கேட்கும் சிரமம் கொண்டவர்கான பயிற்சி) பற்றி உங்களுக்குத் தெரியுமா?
a. ஆம் b. இல்லை c. அறிந்திருக்கவில்லை
ஆம் எனில், விவரங்களைத்தரவும்
____

7. கேட்கும் நிபுணர் (ஆடியோலஜிஸ்ட்) பற்றி உங்களுக்குத் தெரியுமா? (கேட்கும் திறனை சரிபார்ப்பது, காது கருவியை வழங்குதல், பயிற்சி)
a. ஆம் b. இல்லை c. அறிந்திருக்கவில்லை
ஆம் எனில், விவரங்களைத்தரவும்
____

8. பேச்சு மொழி பயிற்சியாளர் பற்றி உங்களுக்குத் தெரியுமா? (பேச்சு, மொழிக்கான பரிசோதனை, பயிற்சிவழங்குதல்)
a. ஆம் b. இல்லை c. அறிந்திருக்கவில்லை
ஆம் எனில், விவரங்களைத்தரவும்
____

9. போக்குவரத்து வசதிகள் / குழந்தைகளுக்கான சுகாதாரப்பாதுகாப்பை (பரிசோதனை/ பயிற்சி/இதர சேவைகள்) பெறுவதற்கான பயணங்கள்
a. எப்போதும் சவாலானது
b. சிலநேரங்களில் சவாலானது
c. சவாலாக இல்லை
d. எனக்கு தெரியாது

10. குழந்தைகள் தொடர்பான சேவைகளின் கிடைக்கும் இடம் (பரிசோதனை/பயிற்சி/இதர சேவைகள்)
a. பொது மருத்துவமனை
b. ஆரம்ப சுகாதார நிலையம்
c. பள்ளி
d. SSA மையம்
e. அங்கன்வாடி
f. வேறுஏதேனும், குறிப்பிடவும்

11. பின்வரும் குழந்தைகள் தொடர்பான பரிசோதனையாளர்/ பயிற்சியாளர்/இதரசேவை வழங்குநரை அணுகுவது மிகவும் கடினமாக உள்ளது
a. பொது மருத்துவமனை
b. ஆரம்ப சுகாதார நிலையம்
c. பள்ளி
d. SSA மையம்
e. அங்கன்வாடி
f. வேறு ஏதேனும்,
குறிப்பிடவும்______________________________________

12. பரிசோதனையாளர்/ பயிற்சியாளர்/இதர சேவை வழங்குநரைப்பார்ப்பது
    a. எப்போதும் சவாலானது
    b. சிலநேரங்களில் சவாலானது
    c. சவாலாக இல்லை
    d. எனக்கு தெரியாது

13. எந்த இடம், பரிசோதனையாளர்/ பயிற்சியாளர்/இதர சேவை வழங்குநரை எளிதாகக் பார்ப்பதற்க்கு சிறந்தது
    a. பொது மருத்துவமனை
    b. ஆரம்ப சுகாதாரநிலையம்
    c. பள்ளி
    d. SSA மையம்
    e. அங்கன்வாடி
    f. வேறுஏதேனும், குறிப்பிடவும்____________________________

14. எந்த இடத்தில் பரிசோதனையாளர்/ பயிற்சியாளர்/இதர சேவை வழங்குநர்களின் இருக்கும் நேரம் குறைவாக உள்ளது
    a. பொதுமருத்துவமனை
    b. ஆரம்பசுகாதாரநிலையம்
    c. பள்ளி
    d. SSA மையம்
    e. அங்கன்வாடி
    f. வேறுஏதேனும், குறிப்பிடவும்

    ____

15. குழந்தைகளுக்கு வீட்டிலேயே சுகாதார சேவைகளை (பரிசோதனை/ பயிற்சி/இதரசேவைகள்) வழங்குதல்
    a. சமூகத்தில் விரும்பத்தக்கது
    b. சமூகத்தில் விருப்பமில்லை.
    விருப்பமில்லைஎன்றால், விவரங்களைவழங்கவும்

    ____
    c. அறிந்திருக்கவில்லை

16. உங்கள் சமூகத்தில் மொபைல்போன்கள் ஏதேனும் சுகாதார சேவைகளைப் (பரிசோதனை/ பயிற்சி/இதரசேவைகள்) பெறப் பயன்படுத்தப்படுகின்றனவா?
    a ஆம், விவரங்களைக் வழங்கவும்

    ____
    b. இல்லை
    c. அறிந்திருக்கவில்லை

17.சமூகத்தில் குறைபாடுகள் உள்ள குழந்தைகளை கண்டறிய மொபைல்போன் அடிப்படையிலான சேவைகள் (பரிசோதனை/ பயிற்சி/இதரசேவைகள்) வழங்கப்பட்டால்

a. மக்கள் உடனடியாக ஏற்றுக் கொள்வார்கள்
b. மக்கள் ஏற்றுக்கொள்வார்கள் ஆனால் நேரம் எடுக்கும்
c. மக்கள் ஏற்றுக்கொள்வார்கள் ஆனால் அதிக விழிப்புணர்வு தேவை
d. மக்கள் ஏற்றுக்கொள்ள மாட்டார்கள்
e. எனக்கு தெரியாது

18. எந்தவொரு நிபுணர் பரிசோதனை மையங்கள் இல்லாத நிலையில், சமூகத்திற்கு அருகில், நிபுணர் இன்டர்நெட் (இணையத்துடன்) மூலம் செவிப்புலன் மற்றும் பேச்சு, மொழி பரிசோதனை நடைமுறைகள் வழங்கப்பட்டால்

a. மக்கள் உடனடியாக ஏற்றுக் கொள்வார்கள்
b. மக்கள் ஏற்றுக் கொள்வார்கள் ஆனால் நேரம் எடுக்கும்
c. மக்கள் ஏற்றுக் கொள்வார்கள் ஆனால் அதிக விழிப்புணர்வு தேவை
d. மக்கள் ஏற்றுக் கொள்ளமாட்டார்கள்
e. எனக்கு தெரியாது

19. எந்தவொரு நிபுணர் மறுவாழ்வு (பயிற்சி) மையங்களும் இல்லாத நிலையில், சமூகத்திற்கு அருகில், நிபுணர் இன்டர்நெட் (இணையத்துடன்) மூலம் செவிப்புலன் மற்றும் பேச்சு மொழிக்கான மறுவாழ்வு வழங்கப்பட்டால்

a. மக்கள் உடனடியாக ஏற்றுக் கொள்வார்கள்
b. மக்கள் ஏற்றுக்கொள்வார்கள் ஆனால் நேரம் எடுக்கும்
c. மக்கள் ஏற்றுக் கொள்வார்கள் ஆனால் அதிகவிழிப்புணர்வு தேவை
d. மக்கள் ஏற்றுக்கொள்ள மாட்டார்கள்
e. எனக்கு தெரியாது

20. சமூகத்தில் நோயறிதல் மற்றும் மறுவாழ்வு (பயிற்சி) வழங்கப்பட்டால், அத்தகைய சேவைகளுக்கு மிகவும் பொருத்தமான இடம் எது

a. பொது மருத்துவமனைகள்
b. பள்ளிகள்
c. அங்கன்வாடி மையங்கள்
d. SSA மையம்
e. தொகுதிக்குள் ஏதாவது இடம்
f. மாவட்டத்திற்குள் ஏதாவது இடம்
g. அருகிலுள்ள முக்கிய நகரங்கள்
